# Engineering *Pseudomonas putida* KT2440 for the production of isobutanol

**DOI:** 10.1002/elsc.201900151

**Published:** 2020-02-18

**Authors:** Robert Nitschel, Andreas Ankenbauer, Ilona Welsch, Nicolas T. Wirth, Christoph Massner, Naveed Ahmad, Stephen McColm, Frédéric Borges, Ian Fotheringham, Ralf Takors, Bastian Blombach

**Affiliations:** ^1^ Institute of Biochemical Engineering University of Stuttgart Stuttgart Germany; ^2^ Ingenza Ltd., Roslin Innovation Centre Charnock Bradley Building, Easter Bush Campus Roslin UK; ^3^ Laboratoire d'Ingénierie des Biomolécules (LIBio) Université de Lorraine Nancy France; ^4^ Microbial Biotechnology, Campus Straubing for Biotechnology and Sustainability Technical University of Munich Straubing Germany

**Keywords:** isobutanol, ketoacid decarboxylase, metabolic engineering, microaerobic, *Pseudomonas putida*

## Abstract

We engineered *P. putida* for the production of isobutanol from glucose by preventing product and precursor degradation, inactivation of the soluble transhydrogenase SthA, overexpression of the native *ilvC* and *ilvD* genes, and implementation of the feedback‐resistant acetolactate synthase AlsS from *Bacillus subtilis*, ketoacid decarboxylase KivD from *Lactococcus lactis*, and aldehyde dehydrogenase YqhD from *Escherichia coli*. The resulting strain *P. putida* Iso2 produced isobutanol with a substrate specific product yield (*Y*_Iso/S_) of 22 ± 2 mg per gram of glucose under aerobic conditions. Furthermore, we identified the ketoacid decarboxylase from *Carnobacterium maltaromaticum* to be a suitable alternative for isobutanol production, since replacement of *kivD* from *L. lactis* in *P. putida* Iso2 by the variant from *C. maltaromaticum* yielded an identical Y_Iso/S_. Although *P. putida* is regarded as obligate aerobic, we show that under oxygen deprivation conditions this bacterium does not grow, remains metabolically active, and that engineered producer strains secreted isobutanol also under the non‐growing conditions.

**Abbreviations**
2‐KIV: 2‐ketoisovalerate
AlsS: acetolactate synthase
BHI: brain–heart infusion
KDC: ketoacid decarboxylase
LB: Lysogeny broth

## 1 INTRODUCTION

Biofuel production from renewable feed stocks is of special importance because of the finite nature of the currently used crude oil derivatives and growing concerns about climate change 1. Isobutanol is an attractive alternative to the employed fossil fuels. It has several advantages such as a higher energy density, compatibility with existing engines, lower vapor pressure and volatility, as well as a lower corrosivity compared to bio‐ethanol 2, 3. Furthermore, isobutanol is used in the chemical industry and can be used to produce the gaseous alkene precursor isobutene 4.

**Figure 1:**
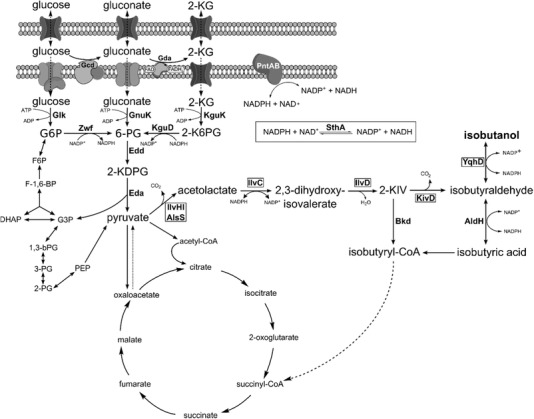
The central metabolism of *P. putida* KT2440 with the Ehrlich pathway. Abbreviations (coding genes are given in brackets): G6P: glucose‐6‐phosphate 2‐KG: 2‐ketogluconate, 2‐K6PG: 2‐keto‐6‐phosphogluconate, 6‐PG: 6‐phosphogluconate, 2‐KDPG: 2‐keto‐3‐deoxy‐6‐phosphogluconate, G3P: glyceraldehyde‐3‐phosphate, 1,3‐bPG: 1,3‐bisphosphoglycerate, 3‐PG: 3‐phosphoglycerate, 2‐PG: 2‐phosphoglycerate, PEP: phosphoenolpyruvate, DHAP: dihydroxyacetone‐phosphate, F‐1,6‐bP: fructose‐1,6‐bisphosphate, F6P: fructose‐6‐phosphate, CoA: co‐enzyme A, Gcd: glucose dehydrogenase (*gcd*), *gad*: gluconate 2‐dehdyrogenase (*gad*), PQQ: pyrroloquinoline quinone, Glk: glucokinase (*glk*), Zwf: glucose‐6‐phosphate 1‐dehydrogenase (*zwf‐1, zwf‐2, zwf‐3*), GnuK: gluconate kinase (*gnuK*), KguD: 2‐6‐phosphoketogluconate reductase (*kguD*), KguK: 2‐ketogluconate kinase (*kguK*), Edd: 6‐phosphogluconate dehydratase (*edd*), Eda: 2‐keto‐3‐deoxy‐6‐phosphogluconate aldolase (*eda*), IlvHI/AlsS: acetolactacte synthase (*ilvHI/alsS*), IlvC: ketolacid reductoisomerase (*ilvC*), IlvD: dihydroxyacid dehydratase (*ilvD*), KivD: ketoacid decarboxylase (*kivD*), Bkd: branched‐chain ketoacid dehydrogenase complex (*bkd*), Yqhd: aldehyde reductase (*yqhD*), AldH: aldehdye dehdyrogenases, PntAB: pyridine nucleotide transhydrogenase (membrane bound) (*pntAB*), SthA: pyridine nucleotide transhydrogenase (soluble) (*sthA*)

Isobutanol can be synthesized via the branched‐chain amino acid biosynthesis and the so‐called Ehrlich pathway to convert pyruvate to isobutanol (Figure 1). The first step in this route is the conversion of two pyruvate molecules to 2‐acetolactate catalyzed by the acetolactate synthase (AlsS), which is usually feedback inhibited by the branched‐chain amino acids l‐valine, l‐leucine, and l‐isoleucine. However, AlsS from *Bacillus subtilis* has been shown to be feedback‐resistant and therefore has been applied for isobutanol production in several studies 5, 6. Then, 2‐acetolactate is reduced to 2,3‐dihydroxyisovalerate and subsequently converted to 2‐ketoisovalerate (2‐KIV) by the ketoacid reductoisomerase IlvC and dihydroxyacid dehydratase IlvD, respectively. Finally, isobutanol is synthesized from 2‐KIV in two more reaction steps of the Ehrlich pathway. The decarboxylation of 2‐KIV to isobutyraldehyde is catalyzed by ketoacid decarboxylases (KDCs) that are not widespread in nature. Especially KivD from *Lactococcus lactis* has been proved as an efficient variant in, e.g. *E. coli* and *C. glutamicum* 5, 7. The last step from isobutyraldehyde to isobutanol requires an aldehyde reductase or alcohol dehydrogenase. A number of NADH and NADPH dependent enzymes are available that catalyze this reaction 8.

Several microorganisms have been engineered for isobutanol production such as *E. coli*, *C. glutamicum*, *B. subtilis*, and yeast such as *Saccharomyces cerevisiae* 5, 7, 9, 10. Although highly efficient *E. coli* and *C. glutamicum* strains have been constructed 6, 7, the relatively low tolerance of most microbial systems against isobutanol hampers commercialization of isobutanol production processes. In contrast, pseudomonads have an intrinsic tolerance against organic compounds and solvents 11, 12 making them promising candidates for isobutanol production.

Among them, *Pseudomonas putida* is a Gram‐negative, saprophytic soil bacterium with a genome size of 6.18 Mbp 13. It has been reported to promote plant growth, prevent plant diseases, and can efficiently remove organic soil pollutants and environmental contaminants 14. *P. putida* features a versatile metabolism using the Entner–Doudoroff pathway for glucose catabolism, shows resistance against oxidative stress conditions, and genetic engineering tools are readily available 15, 16, 17. The carbohydrate substrate spectrum is limited and confined to hexoses 18, however, *P. putida* has been recently engineered to concomitantly consume xylose, cellobiose, and glucose, which are the basic building blocks of the abundant polysaccharides cellulose and hemicellulose 19. As a result of these achievements, *P. putida* has emerged as a promising candidate for industrial biotechnology 20, 21. Recent works have engineered this bacterium for the production of polyhydroxyalkanoates, the nylon precursor *cis,cis*‐muconic acid 22 and aromatic compounds like *p*‐coumaric acid or *trans*‐cinnamate 23, 24. *P. taiwanensis* VLB120 has been applied for the production of phenol 25, 26.

**PRACTICAL APPLICATION**

The relatively low tolerance of most microbial systems against isobutanol hampers commercialization of isobutanol production processes. In contrast, pseudomonads have an intrinsic tolerance against organic compounds and solvents making them promising candidates for isobutanol production. Therefore, we engineered *Pseudomonas putida* KT2440 for the production of this alcohol by preventing product and precursor degradation and increasing the flux from pyruvate toward isobutanol. The achieved overall isobutanol yield is significantly higher compared to other engineered *P. putida* strains; however, rather low compared to tailored *E. coli* and *C. glutamicum* strains. Therefore, this study paths the way to construct more efficient *P. putida* strains for isobutanol production in future studies.

In this study, we engineered *P. putida* for the production of isobutanol from glucose by preventing product and precursor degradation and increasing the flux from pyruvate towards isobutanol. We identified KivD from *Carnobacterium maltaromaticum* as a suitable alternative to KivD from *L. lactis* to drive the decarboxylation of 2‐ketoisovalerate and finally we showed that isobutanol production can also be achieved under oxygen deprivation conditions with this obligate aerobic bacterium.

## 2 MATERIALS AND METHODS

### 2.1 Bacterial strains and plasmids

Bacterial strains, their respective genotype, plasmids, and oligonucleotides used in this study are listed in Table 1.

**Table 1:** Overview of strains, plasmids and oligonucleotides used in this study

Strain, plasmid or oligonucleotide	Relevant characteristic(s) or sequence (5′ → 3′)	Source, reference or purpose
Strains		
* Pseudomonas putida* KT2440	Wild type strain, DSM‐6125, ATCC47054	27
* Carnobacterium maltaromaticum* LMA28		28
* Lactococcus lactis* subsp. c*remoris* MG1363		29
* Corynebacterium glutamicum*	Wild type strain ATCC13032	American type culture collection
* P. putida* GN346	*P. putida* KT2440 Δ*upp*, Δ*pedE*, Δ*pedI*, Δ*pedH*, Δ*aldB‐I*	30
* P. putida* EP1	*P. putida* GN346 Δ*bkdAA*	This work
* P. putida* EP2	*P. putida* EP1 Δ*sthA*	This work
* P. putida* EP3	*P. putida* EP2 Δ*gcd*	This work
* P. putida* Iso1	*P. putida* EP1 + pIP02	This work
* P. putida* Iso2	*P. putida* EP2 + pIP02	This work
* P. putida* Iso3	*P. putida* EP2 + pIP03	This work
* P. putida* Iso4	*P. putida* EP2 + pIP04	This work
* P. putida* Iso5	*P. putida* EP3 + pIP02	This work
* P. putida* Iso6	*P. putida* EP2 + pIP05	This work
Plasmids		
pBB1	pACYC184/pBL1 derivative, chloramphenicol resistance, P_tac_ promoter and *trpA* terminator	31
pSA55	Expression plasmid for *adh2* of *S. cerevisae* and *kivD* of *L. lactis*	5
pBB1 *yqhD*	pBB1 P_tac_ *yqhD*	This work
pBB1 *kivD yqhD*	pBB1 P_tac_ *kivD yqhD*	This work
pNG413.1	pBBR1MCS2 derivative, apramycin resistance, *araC*, P_BAD_, *lacZ*	32
pSEVA231	pBBR1 derivative, kanamycin resistance, mobilizable (*oriT*)	33
pIP01	pSEVA231P_tac_ *kivD yqhD*	This work
pIP02	pNG413 *araC* P_BAD_ *kivD yqhD alsS ilvC ilvD*	This work
pIP03	pIP02, *yqhD* was changed for *adhA* from *L. lactis*	This work
pIP04	pIP02, *yqhD* was changed for *adhA* from *C. glutamicum*	This work
pIP05	pIP02, *kivD* was changed for *kdcA* from *C*. *maltaromaticum*	This work
pEMP04	pSEVA231 P_tac_ *kivD yqhD alsS ilvC ilvD*	Ingenza Ltd.
pEMP012	pEMP04, *yqhD* was changed for *adhA* from *L. lactis*	This work
pEMP013	pEMP04, *yqhD* was changed for *adhA* from *C. glutamicum*	This work
pEMP014	pEMP04, *kivD* was changed for kdcA from *C*. *maltaromaticum*	This work
Oligonucleotide		
yqhd1	AACTGCAGAACCAATGCATTGGAGGAGACACAACA TGAACAACTTTAATCTGCACACCCCAACC	Construction of pBB1yqhd, PstI site underlined
yqhd2	CCGCTCGAGAAAGCTTAGCGGGCGGCTT CGTATATACG	Construction of pBB1yqhd, XhoI site underlined
kivd1	TCCCCCCGGGAGGAGACACAACATGTATACAGTAGGAG ATTACCTAT	Construction of pBB1 kivd yqhd, XmaI site underlined
kivd2	CCAATGCATTGGTTCTGCAGTTTTATGATTTATTTTGTTC AGCAAAT	Construction of pBB1 kivd yqhd, PstI site underlined
bkdaa1	CTGGATCCCATTCAGACCTCCATGACC	Deletion of *bkdAA*
bkdaa2	CGGCCGCTTCAGAGCTCACATGAGATGAACGA CCACAAC	Deletion of *bkdAA*
bkdaa3	TGTTGTGGTCGTTCATCTCATGTGAGCTCTG AAGCGGC	Deletion of *bkdAA*
bkdaa4	GCTTGTCGACCCGTCGTCACTGCCGTAG	Deletion of *bkdAA*
bkdaagc1	GTACCGACGATGCCGCT	Verification of *bkdAA* deletion
bkdaagc2	GCCGTGCCACTAAGATGTAG	Verification of *bkdAA* deletion
stha1	GCCGCTTTGGTCCCGGATCCACAGCATCCAGTACGT CCGC	Deletion of *sthA*
stha2	GTTGAAATCGGTCTCTCCGACCTGAACGCCGCGCACA TTAAC	Deletion of *sthA*
stha3	GTTAATGTGCGCGGCGTTCAGGTCGGAGAGACCGATTT CAAC	Deletion of *sthA*
stha4	TTGCATGCCTGCAGGTCGACTGGTTGGGCAAACCCTGC TTGG	Deletion of *sthA*
sthagc1	ATGGCTATTCGACGCTGCTG	Verification of *sthA* deletion
sthagc2	ACTATGGCTGCGAACTGCTG	Verification of *sthA* deletion
gcd1	GCCGCTTTGGTCCCGGATCCTGACCTTGAGTTGTTCC TTG	Deletion of *gcd*
gcd2	GACCTGACGGAGAACCTACATTAGCCGAGT AAGCGACAC	Deletion of *gcd*
gcd3	GTGTCGCTTACTCGGCTAATGTAGGTTCTCCGTCA GGTC	Deletion of *gcd*
gcd4	TTGCATGCCTGCAGGTCGACGACAACATCAGCAACG ACC	Deletion of *gcd*
gcdgc1	GGGATGGGTTTCAATGGTTC	Verification of *gcd* deletion
gcdgc2	GGCACAAGATGTTCTCAAGG	Verification of *gcd* deletion
png1	AGCTCTAAGGAGGTTATAAAAACATATGTATACAGTAGG AGATTACC	Construction of pIP02, pIP03, pIP04 and pIP05
png2	GAGAATAGGAACTTCGAACTGCAGGTCGACTCAGAGG CCTTCCAGC	Construction of pIP02, pIP03 and pIP04
png3	AGCTCTAAGGAGGTTATAAAAACATATGTACACTGTT GGAAATTATTTGTTA	Construction of pIP05
pbb1	TCGGAGCTCCGCGAATTGCAAGCTGATCCG	Construction of pIP01, SacI site underlined
pbb2	ATCGGATCCCTTAGCGGGCGGCTTCGTAT	Construction of pIP01, BamHI site underlined
kdca1	TTGCTAAACAAAATTCATAAAACTGCAGAACCAATGC	Amplification of *kdcA* gene
kdca2	AATGCATTGGTTCTGCAGTTTTATGAATTTTGTTTAGC AAAGACTTTC	Amplification of *kdcA* gene
p41	TTGCTAAACAAAATTCATAAAACTGCAGAACCAATG CATTG	Construction of pEMP014
p42	TAATTTCCAACAGTGTACATGTTGTGTCTCCTCCCGG	Construction of pEMP014
p43	TCATTGATTTTACTAAATAAGCCAGGAGGACAGCTAT	Construction of pEMP012
p44	CGTACTACTGCTGCTTTCATGTTGTGTCTCCTCCAATGC	Construction of pEMP012
p45	GTGTGGCGATTCGTTTCTAAGCCAGGAGGACAGCTA TGAC	Construction of pEMP013
p46	TGGGGTGCAGCAGTGGTCATGTTGTGTCTCCTCCAA TGCATTG	Construction of pEMP013
adha1	GCATTGGAGGAGACACAACATGAAAGCAGCAGTA GTACG	Amplification of *adhA* gene from *L. lactis*
adha2	GTCATAGCTGTCCTCCTGGCTTATTTAGTAAAATC AATGACCATCC	Amplification of *adhA* gene from *L. lactis glutamicum*
adha3	TGCATTGGAGGAGACACAACATGACCACTGCTGCACC	Amplification of *adhA* gene from *C. glutamicum*
adha4	GTCATAGCTGTCCTCCTGGCTTAGAAACGAATCG CCACACG	Amplification of *adhA* gene from *C. glutamicum*
alss1	GAGGAAAGCGGCCGCGCTCTTCGGGGCGGAGCTTGTTG	Construction of pEMP04, NotI site underlined
alss2	TTAGATCTCGAGGCTCTTCGGGCCTAGAGAGCTTTCG TTTTCATG	Construction of pEMP04, XhoI site underlined
ilvc1	GAGGAAGCGGCCGCGCTCTTCGAAGAAAGTCGCCATCATC	Construction of pEMP04, NotI site underlined
ilvc2	TTAGATCTCGAGGCTCTTCGGGCTTAGTTCTTGGTC TTGTCGAC	Construction of pEMP04, XhoI site underlined,
ilvd1	GAGGAAGCGGCCGCGCTCTTCGCGGCGCCCGTG	Construction of pEMP04, NotI site underlined
ilvd2	TTAGATCTCGAGGCTCTTCGGGCTCAGAGGCCTTCCAG	Construction of pEMP04, XhoI site underlined
pip011	GAGGAAGCGGCCGCGCTCTTCGCGTGACTGGGAAAACCC TGGCGACTAGTCTTGGACTC	Construction of pEMP04, NotI site underlined
pip012	TTAGATCTCGAGGCTCTTCGGGCTTAGCGGGCGGCTTCG TATATACGGCGGCTGA	Construction of pEMP04, XhoI site underlined

### 2.2 Media and culture conditions

*E. coli* DH5α was grown aerobically in Lysogeny broth (LB) complex medium containing 10 g/L tryptone, 5 g/L yeast extract, and 10 g/L NaCl 34 at 37ºC as 5 mL cultures in glass test tubes on a rotary shaker at 120 rpm (Infors AG, Bottmingen, Switzerland). *C. maltaromaticum* and *L. lactis* were grown in brain–heart infusion (BHI) broth (Carl Roth GmbH & Co. KG, Karlsruhe, Germany) at 30°C on a rotary shaker at 120 rpm. For longtime storage, *P. putida* was kept as 30% (*w/v*) glycerol stock at −70°C and was streaked out for cultivation on LB solid medium with 15 g/L agar. The first preculture of *P. putida* was prepared by inoculation of 5 mL LB medium in a test tube with a single colony. The culture was cultivated at 30°C on a rotary shaker (Edmund Bühler GmbH, Bodelshausen, Germany) at 175 rpm overnight and used to inoculate, a second overnight preculture to an optical density at 600 nm (OD_600_) of 0.01–0.02 in 50 mL DeBont minimal medium (pH 7) 35, which was supplemented with 5.4 g/L glucose and 0.5 g/L yeast extract. Cells from the second preculture were harvested by centrifugation (4500 × *g*, 15 min, 4°C), resuspended in DeBont medium, and used to inoculate 50 mL DeBont medium, to an OD_600_ of about 0.1–0.2. The main culture was supplemented with 5.4 g/L glucose, 0.5 g/L isobutanol, or 2.9 g/L 2‐ketoisovalerate, respectively. The second pre‐ and main cultures were performed in 500 mL baffled Erlenmeyer flasks filled with 50 mL medium on a rotary shaker at 175 rpm at 30°C. Micro‐aerobic shaking flask cultivations were carried out in sealed 100 mL Müller‐Krempel bottles as 25 mL cultures that were inoculated to an OD_600_ of 15–20. To obtain sufficient biomass, the second preculture was performed in 100 mL LB medium in a 500 mL baffled Erlenmeyer flask that was cultivated on a rotary shaker (175 rpm) overnight at 30°C. Cells from the second preculture were harvested by centrifugation (4500 × *g*, 15 min, 4°C) and resuspended in 25 mL DeBont minimal medium (pH 7) supplemented with 5.4 g/L glucose and 15 g/L 3‐morpholino‐propanesulfonic acid. To induce plasmid‐based gene expression, 0.2% (*w/v*) l‐arabinose was supplemented. For plasmid‐bearing strains, 50 µg/mL kanamycin or 50 µg/mL apramycin were added to the medium.

### 2.3 Recombinant DNA work

Standardized cloning procedures such as PCR and DNA restrictions were carried out according to Sambrook and Russell, 2001. Plasmids were isolated from 5 mL liquid cultures using the E.Z.N.A.^®^ Plasmid Mini Kit (Omega Bio‐tek, Inc., Norcross, USA) following manufacturer's instructions. PCR fragments were purified with the NucleoSpin^®^ Gel and PCR Clean‐up Kit (Macherey‐Nagel GmbH & Co. KG, Düren, Germany) according to the manufacturer's instructions. Chromosomal DNA of *E. coli* MG1655, *P. putida, C. maltaromaticum*, and *L. lactis* was isolated using the Nucleospin^®^ Microbial DNA Kit (Macherey‐Nagel GmbH & Co. KG, Düren, Germany) following the protocol of the manufacturer. Electrocompetent cells were prepared for *E. coli* and *P. putida* as described previously 53, 54. *E. coli* DH5α and *P. putida* strains were electroporated with an Eporator (Eppendorf AG, Hamburg, Germany) at 2.5 kV with 600 Ω resistance. All enzymes for recombinant DNA work were obtained from Thermo Fisher Scientific Inc. (Darmstadt, Germany) and oligonucleotides were synthesized by biomers.net GmbH (Ulm, Germany, listed in Table 3).

### 2.4 Plasmid construction

*yqhD* was amplified from genomic DNA of *E. coli* MG1655 using the primers yqhd1/yhqd2, digested with PstI/XhoI and ligated into PstI/XhoI‐digested pBB1 yielding pBB1 yqhD. *kivD* was subsequently added before *yqhD*, amplified from pSA55 with the primer pair kivd1/kivd2, digested with PstI/XmaI, and ligated into PstI/XmaI‐digested pBB1 yqhd creating plasmid pBB1 kivD yqhD. P_tac_, *kivD*, and *yqhD* were amplified from plasmid pBB1 kivD yqhD using the primers pbb1/pbb2. The resulting PCR fragment was digested with BamHI/SacI and subsequently ligated into BamHI/SacI‐digested pSEVA231 to create plasmid pIP01. Plasmid pEMP04 was constructed using the inABLE DNA assembly method from Ingenza Ltd. The *B. subtilis* *alsS* and *P. putida* *ilvC* and *ilvD* genes were amplified using primer pairs alss1/alss2, ilvc1/ilvc2, and ilvd1/ilvd2, respectively. Additionally, a 5′ truncated version of pIP01 was amplified using primer pair pip011/pip012. The PCR products were digested using SapI and annealed oligonucleotides were ligated at each terminus.  Ligation of the oligonucleotides results in the generation of 5′ and 3′ 16 nt single stranded overhangs that are complementary between fragments resulting in the DNA assembling in the predefined order. The genes of pEMP04 were amplified using the primers png1/png2 and cloned by Gibson Assembly 36 into NdeI/SalI‐digested pNG413.1 yielding plasmid pIP02. *kdcA* from *C. maltaromaticum* LMA28, *adhA* from *L. lactis* MG1363, and *adhA* from *C. glutamicum* was amplified using the respective genomic DNA with the primers kdca1/kdca2, adha1/adha2, and adha3/adha4 and cloned together via Gibson Assembly with a PCR fragment from pEMP04 that was amplified with the primers p41/p42, p43/p44, or p45/p46 to construct plasmid pEMP014, pEMP012, and pEMP013. To exchange P_tac_ with *araC* P_BAD_ the genes of pEMP012, pEMP013 and pEMP014 were amplified using the primers png1/png2 for pEMP012/013 and png3/png2 for pEMP014 and cloned by Gibson Assembly into NdeI/SalI‐digested pNG413.1, constructing the plasmids pIP03, pIP04, and pIP05.

### 2.5 Determination of **μ** and *Y*_X/S_

Growth rates were determined by linear regression of ln(OD_600_) plotted against time (in hours) during the exponential growth phase. Biomass yields *Y*_X/S_ (g/g) were calculated by linear regression of the biomass concentration c_x_ (g/L) plotted against the respective glucose concentration (g/L) during the exponential growth phase.

### 2.6 Construction of *P. putida* deletion mutants

Chromosomal deletions in *P. putida* were carried out using the 5‐fluorouracil (5‐FU)/*upp* counterselection system 37. Deletions of the *bkdAA* gene (encoding the α‐subunit of the ketoacid dehydrogenase complex), the *sthA* gene (encoding soluble transhydrogenase) and the *gcd* gene (encoding glucose dehydrogenase) were performed using the integration vector pJOE6261.2. The flanking regions (about 500 bp) of each gene were amplified by PCR from chromosomal DNA of *P. putida* using the primer pairs bkdaa1/bkdaa2 and bkdaa3/bkdaa4, stha1/stha2, and stha3/stha4, gcd1/gcd2 and gcd3/gcd4. The two respective PCR fragments were purified and cloned into SalI/BamHI‐restricted pJOE6261.2 by Gibson Assembly. Finally, the assembly mix was used to transform *P. putida* by electroporation. The first selection was carried out on LB agar with 50 µg/L kanamycin and a kanamycin‐resistant clone was afterward grown in liquid LB medium for 24 h. The second recombination event was induced by plating cells on LB agar with 50 µg/L 5‐FU. Deletion mutants were identified by colony PCR using the primer pairs bkdaagc1/bkdaagc2, sthagc1/sthagc2, and gcdgc1/gcdgc2, respectively.

### 2.7 Analytics

Biomass formation was measured by determination of the OD_600_ (Ultrospec 10, GE Healthcare, USA) at specific time points. The cell dry weight (g_CDW_/L) was correlated to the OD_600_ in several independent cultivations with a correlation factor of 0.346 g_CDW_/L per OD (data not shown). Shaking flasks were sampled directly in the incubator using an injection syringe (100 Sterican^®^, 0.80 × 120 mm, B.Braun, Melsungen, Germany). For the determination of isobutanol, 2‐KIV, 2‐ketogluconate (2‐KG), and glucose concentrations, 2 mL of the main culture was harvested by centrifugation (12 100 × *g*, 5 min, room temperature (RT)) and the supernatant was analyzed via HPLC. Glucose concentrations were measured enzymatically with a test kit from r‐biopharm (r‐biopharm AG, Darmstadt, Germany).

### 2.8 HPLC metabolite quantification

Isobutanol, 2‐KIV and 2‐KG were measured with a Agilent 1200 series HPLC system equipped with a Rezex ROA organic acid H (8%) column (300 by 7.8 mm, 8 µm; Phenomenex) protected by a Phenomenex guard column carbo‐H (4 by 3.0 mm inside diameter) 38. Samples and standards were treated with a phosphate precipitation protocol before HPLC measurements. More precisely, 500 µL of sample volume was mixed with 45 µL 4 M NH_3_ and 50 µL 1.2 M MgSO_4_ followed by 5 min incubation at RT and centrifugation for 5 min at 7000 × *g*. Pellets were discarded and the supernatant was mixed with 500 µL 0.1 M H_2_SO_4_, incubated for 15 min at RT, and centrifuged for 15 min at 7000 × *g*. The resulting supernatant was used for HPLC injection with an injection volume of 10 µL. Separation was carried out under isocratic conditions at 50°C column temperature for 60 min with 5 mM H_2_SO_4_ as the mobile phase at a constant flow rate of 0.4 mL/min. Detection of isobutanol, 2‐KIV, and 2‐KG was achieved with a refractive index detector at 32°C. Quantification of all analytes was done with a 7‐point calibration curve for each component as an external reference standard.

## 3 RESULTS

### 3.1 Preventing product and precursor degradation

**Figure 2:**
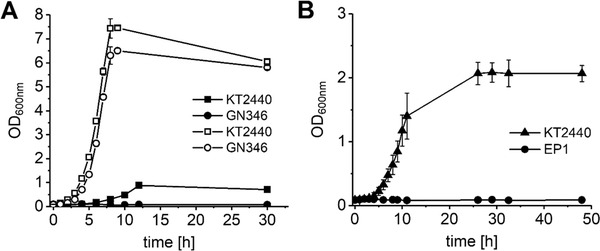
(A) Growth of *P. putida* KT2440 and *P. putida* GN346 in DeBont minimal medium containing 0.5 g/L isobutanol (filled symbols) or 5.4 g/L glucose (open symbols). (B) Growth of *P. putida* and *P. putida* EP1 in DeBont minimal medium containing 2.9 g/L 2‐ketoisovalerate. Experiments were performed in triplicates and error bars represent the corresponding standard deviation

*Pseudomonads* are well‐known for their ability to degrade a variety of organic substances to utilize them as carbon and energy sources 21. Since the genomic repertoire provides annotated routes for the degradation of isobutanol and 2‐ketoisovalerate (Figure 1), we initially characterized growth on both compounds (Figure 2). *P. putida* showed exponential growth on isobutanol with a μ of 0.27 ± 0.01 h^−1^ as well as on 2‐KIV with a μ of 0.33 ± 0.01 h^−1^ that is 52% of the growth rate on glucose (Figure 2A,B). Recently, several enzymes involved in *n*‐butanol degradation were identified 39 and Simon et al. 30 constructed *P. putida* Δ*upp* Δ*pedE* Δ*pedI* Δ*pedH* Δ*aldB‐I* (*P. putida* GN346) to inactivate two alcohol dehdyrogenases (PedE, PedH) and two aldehyde dehydrogenases (PedI, AldB‐I) and showed that the introduced deletions prevented *n*‐butanol consumption. Accordingly, *P. putida* GN346 was unable to utilize isobutanol as sole carbon and energy source (Figure 2A).

*P. putida* possesses a branched chain ketoacid dehydrogenase (BCKDH) complex that converts 2‐ketoacids to the respective decarboxylated CoA‐derivatives 40, 41 which are, after further conversion steps, funneled into the TCA cycle. To prevent the consumption of the precursor 2‐KIV, we inactivated the α‐subunit of the BCKDH by deletion of the *bkdAA* gene in *P. putida* GN346. In contrast to the wild‐type, the resulting strain *P. putida* EP1 was unable to grow on 2‐KIV as carbon source (Fig. 2B), and therefore was used as basis for further strain engineering.

### 3.2 Engineering *P. putida* for isobutanol production

To drain the carbon from pyruvate to 2‐KIV, we constructed a plasmid harboring the *alsS* gene encoding the acetolactate synthase from *Bacillus subtilis*, which is not feedback inhibited by branched chain amino acids, and the native *ilvCD* genes encoding the ketolacid reductoisomerase and dihydroxyacid dehydratase (Figure 1). For the conversion of 2‐KIV to isobutanol, we additionally cloned *kivD* encoding the KDC from *Lactococcus lactis* and *yqhD* encoding an aldehyde reductase from *E. coli* (Fig. 1). AlsS, KivD, and YqhD were previously applied for isobutanol production in other hosts such as *C. glutamicum* and *E. coli* 5, 7. The resulting plasmid pIP02 expresses all cloned genes under control of the l‐arabinose inducible P*_BAD_* promoter and was used to transform *P. putida* EP1 yielding *P. putida* Iso1. In minimal medium with glucose, *P. putida* Iso1 showed a µ = 0.56 ± 0.02. Although the *Y*_X/S_ was reduced by 25% compared to the wild‐type, no isobutanol was produced during the cultivation (Table 2).

**Table 2:** Overview of growth, 2‐ketogluconate (2‐KG) and isobutanol production of *P. putida* and its engineered derivatives

Strain	μ [h^−1^]	*Y*_X/S_ [g/g]	*Y*_2‐KG/S_ [mg/g_GLC_]	*Y*_Iso/S_ [mg/g_GLC_]
KT2440	0.62 ± 0.01	0.40 ± 0.01	0	0
GN346	0.59 ± 0.01	0.39 ± 0.01	0	0
Iso1	0.56 ± 0.02	0.30 ± 0.01	0	0
Iso2	0.25 ± 0.01	0.13 ± 0.01	438 ± 20	22 ± 2
Iso3	0.14 ± 0.01	0.06 ± 0.01	833 ± 100	13 ± 1
Iso4	0.19 ± 0.01	0.09 ± 0.02	771 ± 15	14 ± 0.0
Iso5	0.18 ± 0.02	0.28 ± 0.01	0	0
Iso6	0.28 ± 0.01	0.10 ± 0.01	633 ± 39	21 ± 1

The synthesis of isobutanol from glucose requires 2 mol NAD(P)H per mol isobutanol. The reduction of acetolactate is catalyzed by NADPH‐dependent ketolacid reductoisomerase (IlvC), while the conversion of isobutyraldehyde to isobutanol can be catalyzed by NAD(P)H‐dependent aldehyde/alcohol dehydrogenases such as YqhD (Figure 1). Since YqhD is NADPH‐dependent, the engineered iosobutanol pathway should consume 2 mol NADPH per mol isobutanol. *P. putida* possesses a membrane‐bound and a soluble transhydrogenase. The latter is encoded by the *sthA* gene 42 and has in *E. coli* been reported to favor the re‐oxidation of NADPH to NADP^+^ under reduction of NAD^+^ to NADH 43, 44.

**Figure 3:**
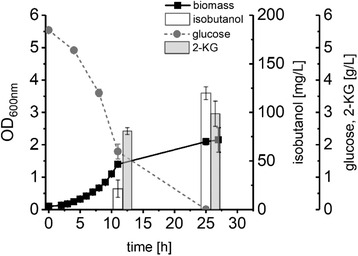
Growth (black circles), glucose consumption (grey circles), isobutanol production (white bars) and 2‐KG formation (grey bars) of *P. putida* Iso2 in DeBont minimal medium containing glucose. Experiments were performed in triplicates and error bars represent the corresponding standard deviation

To test whether the inactivation of the soluble transhydrogenase is beneficial for isobutanol production, we deleted the *sthA* gene in *P. putida* EP1, yielding *P. putida* EP2 which was transformed with the plasmid pIP02. The resulting strain *P. putida* Iso2 showed in minimal medium containing 5.4 g/L glucose, a growth rate of 0.26 ± 0.01 h^−1^, a *Y*_X/S_ of of 0.13 ± 0.01 g/g, and produced 438 ± 20 mg/g_GLC_ 2‐KG and for the first time isobutanol with a *Y*_Iso/S_ of 22 ± 2 mg/g_GLC_ (Table 2, Figure 3). We also replaced the aldehyde reductase gene *yqhD* on the overexpression plasmid pIP02 with the *adhA* genes encoding NADH‐dependent alcohol dehydrogenase variants from *Lactococcus lactis* and *Corynebacterium glutamicum*, respectively. The plasmids pIP03 and pIP04 were used to transform *P. putida* EP2, yielding *P. putida* Iso3 and Iso4, which were characterized in minimal medium with glucose (Table 2). Both strains showed reduced growth rates and about 40% lower product yields compared to *P. putida* Iso2. All engineered strains with deletion of *sthA* converted 40% to 83% of the available glucose into 2‐KG that was secreted into the culture broth (Table 2). To avoid 2‐KG secretion and to improve isobutanol production, we constructed *P. putida* EP3 by deletion of the *gcd* gene encoding periplasmatic glucose dehydrogenase in *P. putida* EP2. To construct *P. putida* Iso5, *P. putida* EP3 was transformed with the plasmid pIP02. In fact, *P. putida* Iso5 did not secrete any 2‐KG, however, inactivation of GCD also abolished isobutanol production completely (Table 2).

### 3.3 Ketoacid decarboxylase from *Carnobacterium maltaromaticum* is suitable for isobutanol production

The key enzyme for isobutanol production via the Ehrlich pathway is ketoacid decarboxylase (KDC) converting 2‐KIV to isobutyraldehyde (Figure 1). So far, only KDC from *L. lactis* has been proven as suitable variant that efficiently catalyzes this reaction 2, 5. Recently, the genome sequence of *Carnobacterium maltaromaticum* LMA28 28 was published that harbors a gene encoding a putative KDC. KDC from *C. maltaromaticum* shows 54% identity to the KDC enzyme from *L. lactis*. To test the suitability of KDC from *C. maltaromaticum* for isobutanol production, we replaced the *kivD* gene on plasmid pIP02 with the respective gene from *C. maltaromaticum*. Plasmid pIP05 was transformed into *P. putida* EP2 and the resulting strain *P. putida* Iso6 was characterized. *P. putida* Iso6 showed a growth rate of 0.28 ± 0.01 h^−1^, a *Y*_X/S_ of of 0.10 ± 0.01 g/g. and produced 633 ± 39 mg/g_GLC_ 2‐KG. Furthermore, *P. putida* Iso6 secreted as much isobutanol as *P. putida* Iso2 with a *Y*_Iso/S_ of 21 ± 1 mg/g_GLC_ (Table 2) showing that KDC from *C. maltaromaticum* LMA28 is a useful alternative to KDC from *L. lactis*.

### 3.4 Microaerobic isobutanol production in *P. putida*

*P. putida* is regarded as an obligate aerobic bacterium 18. However, since the implementation of the synthetic isobutanol pathway theoretically enables a closed redox balance, we tested the capabilities of our engineered *P. putida* strains to produce isobutanol from glucose in a zero‐growth bioprocess under oxygen deprivation conditions 45. Therefore, we inoculated *P. putida* WT and Iso2–6 to an OD_600_ of 15–20 in closed bottles filled with minimal medium containing 5.4 g/L glucose and characterized substrate consumption and (by‐) product formation (Table 3). In the micro‐aerobic environment *P. putida* WT showed no growth, but remained metabolically active and consumed the glucose that was converted to 2‐KG. With the exception of *P. putida* Iso5, all other engineered strains consumed glucose and produced isobutanol. *P. putida* Iso6 showed the best performance under oxygen deprivation conditions. Compared to the WT the *q*_S_ was reduced by 37% and *P. putida* Iso6 produced about 10% less isobutanol compared to the aerobic shaking flask experiments (Table 3).

**Table 3:** Overview of engineered *P. putida* strains cultivated under oxygen deprivation conditions

Strain	Y_Iso/S_ [mg/g_GLC_]	qs [g g^−1^ h^−1^]	Y_2‐KG/S_ [mg/g_GLC_]
KT2440	0	0.11 ± 0.01	42 ± 32
Iso2	9 ± 1	0.14 ± 0.01	120 ± 9
Iso3	5 ± 2	0.12 ± 0.01	183 ± 8
Iso4	4 ± 1	0.13 ± 0.01	193 ± 12
Iso5	0	0.01 ± 0.00	0
Iso6	19 ± 2	0.07 ± 0.01	397 ± 14

## 4 DISCUSSION

*P. putida* is an emerging host for industrial biotechnology 46, 47, 48. However, this bacterium is also known to efficiently metabolize a broad range of substrates including amino and organic acids and alcohols 21. As shown here, *P. putida* grows rapidly on isobutanol as well as on its precursor 2‐KIV. Although *P. putida* KT2440 possesses four aldehyde dehydrogenases and about 10 alcohol dehydrogenases, Simon et al. 30 showed that deletion of the two alcohol dehydrogenase genes *pedE* and pedH and the two aldehyde dehydrogenases genes *pedI* and *aldB‐I* is sufficient to prevent *n*‐butanol degradation. Accordingly, we found that this strain background also prevents growth on the branched‐chain alcohol isobutanol. *P. putida* possesses a branched chain ketoacid dehydrogenase complex that converts 2‐ketoacids to the respective decarboxylated CoA‐derivatives 40, 41. As expected and also observed for *P. taiwanensis* VLB120 41, inactivation of the BCKDH abolished growth on 2‐ketoisovalerate. To avoid auxotrophies, we relinquished the inactivation of the l‐valine forming transaminase IlvE, the 2‐isopropylmalate synthase LeuA and the 2‐ketoisovalerate hydroxymethyltransferase PanB as has been applied to improve isobutyric acid production with *P. taiwanensis* strain VLB120 41.

Since AHAIR is usually NADPH‐dependent, the synthesis of one molecule of isobutanol either requires two molecules of NADPH or one NADH plus one NADPH molecule depending on the applied alcohol/aldehyde dehydrogenase variant for the reduction of isobutyraldehyde to isobutanol. Optimization of NAD(P)H availability has already been shown to be a crucial factor for isobutanol production with other hosts such as *E. coli* and *C. glutamicum* 6, 49. Recently, Nikel et al. 16 showed that *P. putida* cells growing on glucose exhibit a slight catabolic overproduction of reducing power and run a biochemical cycle that favors NADPH formation. Therefore, we applied in our experiments the broad‐substrate range NADPH‐dependent aldehyde reductase YqhD 50, which has also been successfully applied for isobutanol production with *E. coli* 8. However, expression of the synthetic pathway in *P. putida* Iso1 to channel pyruvate toward isobutanol did not result in isobutanol production from glucose. Similar to *E. coli*, *P. putida* possesses a membrane bound (PntAB) and a soluble transhydrogenase (SthA) to balance the overall redox state of the cell (Figure 1). SthA has in *E. coli* been reported to favor the oxidation of NADPH to NADP^+^, accompanied with the reduction of NAD^+^ to NADH 43, 44. To improve NADPH availability, we inactivated SthA that resulted in isobutanol formation in *P. putida* Iso2 under aerobic conditions. Accordingly, expression of two *adhA* genes encoding NADH‐dependent alcohol dehydrogenases from *L. lactis* and *C. glutamicum*, which have previously been shown to be suitable for isobutanol production 7, 8, instead of YqhD, led to significantly reduced isobutanol yields in the Δ*sthA* background (Table 2).

Inactivation of SthA resulted in isobutanol production, however, also in the secretion of significant amounts of 2‐KG. In *P. putida* a majority of the glucose is converted in the periplasm by glucose dehydrogenase (Gcd) to gluconate, which is transported to the cytoplasm and activated by the gluconate kinase to feed the Entner–Doudoroff pathway with 6‐phosphogluconate. Usually, only a small fraction of gluconate is converted in the periplasm by gluconate dehydrogenase to 2‐KG, which is subsequently transported into the cytoplasm to finally form 6‐phosphogluconate via 2‐KG kinase and 2‐ketogluconate‐6‐P reductase 16. Since deletion of *gcd* abolished 2‐KG production completely, the synthesis of this molecule occurs solely in the periplasm via the described route. The accumulation of 2‐KG in the culture broth indicates a transport inhibition of gluconate and/or 2‐KG from the periplasm to the cytoplasm by an unknown mechanism and/or an inhibition or limitation of the ATP‐dependent conversion to the phosphorylated derivatives. The latter might result as consequence of a perturbed redox state due to the inactivated transhydrogenase SthA.

*P. putida* is an obligate aerobic bacterium, however, in a bioelectrochemical system *P. putida* was metabolically active under anoxic conditions when an electron mediator was applied for redox balancing in a high‐yield 2‐KG production system 51, 52. Since isobutanol synthesis enables regeneration of NAD(P)^+^, we cultivated *P. putida* WT and the engineered derivatives under microaerobic conditions. All strains showed no growth (data not shown) but with the exception of *P. putida* Iso5, remained metabolically active and *P. putida* Iso2‐4 and 6 also secreted 2‐KG, isobutanol, and further unidentified products. However, according to the zero‐growth the *q*_S_ values are low compared to aerobic conditions (e.g. for *P. putida* WT 0.11 vs. 1.55 g g^−1^ h^−1^) . The capability of *P. putida* to remain metabolically active opens the possibility to develop dual‐phase production processes that comprise an aerobic growth phase for rapid biomass formation and a micro‐aeobic or anaerobic production phase 45.

This study paths the way to construct more efficient *P. putida* strains for isobutanol production in future studies. The overall isobutanol yield is significantly higher compared to other engineered *P. putida* strains 41, however, rather low compared to tailored *E. coli* and *C. glutamicum* strains 49. Product and precursor degradation can be prevented by the presented deletions in this study, however, improving NAD(P)H and pyruvate availability 49 will be crucial to achieve high‐yield isobutanol production strains.

## CONFLICT OF INTEREST

The authors have declared no conflict of interest.
